# Supplementary material for: Low frequency of community-acquired bacterial co-infection in patients hospitalized for COVID-19 based on clinical, radiological and microbiological criteria: a retrospective cohort study
Source: Antimicrob Resist Infect Control. 2021 Oct 30;10:155. doi: 10.1186/s13756-021-01024-4 (PMC8556861; doi:10.1186/s13756-021-01024-4)
Supplement: Supplementary file 1 — Additional file 1. Parameters used by the expert panel to categorize patients as having unlikely, possible or probable community-acquired bacterial co-infection. [file 13756_2021_1024_MOESM1_ESM.docx]

**Additional file 1.** **Parameters used by the expert panel to categorize patients as having unlikely,**

**possible or probable community-acquired bacterial co-infection**

|  | **Co-infection less likely** | **Co-infection more likely** |
| --- | --- | --- |
| Clinical parameters | - CRP < 100 mg/L; or  - No CRP decrease after initiation of antibiotics; or  - Satisfactory clinical response in absence of or <3 days of antibiotics. | -CRP decrease after initiation of antibiotics; or  -Clinical deterioration in absence of antibiotics. |
| Radiology | -Chest CT without consolidations (ground glass opacities only), or;  -Chest CT with consolidations not consistent with bacterial infection^1^. | -Large, lobar or unilateral consolidations on chest CT. |
| Microbiology | -Negative test results^2^; or  -Positive test results due to contamination, colonization or extra-pulmonary infection. | -Positive test results not due to contamination, colonization or extra-pulmonary infection. |

Patients without any respiratory symptoms and without altered mental status, and patients with C-

reactive protein (CRP) below 100 mg/L in combination with chest CT findings not consistent with co-

infection and negative microbiological test results were categorized as unlikely co-infection. For all

remaining patients categorization was performed by the expert panel based on individual patients‘

clinical, radiological and microbiological findings.

^1^Chest CT reports that described consolidations which were multifocal, crescent-shaped or

had round and/or oval morphology were classified as not consistent with co-infection. ^2^Culture, PCR

or urinary antigen tests.

CT: Computed tomography (CT) scan; CRP: C-reactive protein
