# Supplementary material for: Low frequency of community-acquired bacterial co-infection in patients hospitalized for COVID-19 based on clinical, radiological and microbiological criteria: a retrospective cohort study
Source: Antimicrob Resist Infect Control. 2021 Oct 30;10:155. doi: 10.1186/s13756-021-01024-4 (PMC8556861; doi:10.1186/s13756-021-01024-4)
Supplement: Supplementary file 2 — Additional file 2. Microbiological tests and identified bacterial pathogens within 72 h of admission. [file 13756_2021_1024_MOESM2_ESM.docx]

**Additional file 2**. **Microbiological tests and identified bacterial pathogens within 72h of admission**

| Diagnostic test | Performed ≤72h, | Positive result | Identified micro-organisms |
| --- | --- | --- | --- |
| Sputum culture | 88 (31) | 9/88 (10) | *7 x Staphylococcus aureus* |
|  |  |  | *1 x Proteus mirabilis* |
|  |  |  | *1 x Haemophilus influenzae* |
|  |  |  | *1 x Moraxella catarrhalis* |
|  |  |  | *1 x Klebsiella pneumoniae* |
| Blood culture* | 232 (83) | 2/232 (1) | *1 x Acinetobacter lwoffi* |
|  |  |  | *1 x Escherichia coli* |
| Broncho-alveolar lavage culture | 4 (1) | 1/4 (25) | *1 x Burkholderia cenocepacia* |
| Urinary pneumococcal antigen test | 104 (37) | 2/104 (2) | *2 x Streptococcus pneumoniae* |
| Atypical pathogens - Urinary legionella antigen test - Mycoplasma pneumonia PCR  - Chlamydia pneumoniae and Chlamydia psittaci PCR | 60 (21) 35 (12)  30 (11)  29 (10) | 1/60 (1.7) 0/35 (0)   1/30 (3) 0/29 (0) | 1 x *Mycoplasma pneumoniae* |
| Any microbiological test performed | 251/281 (89) | 15/251 (6) |  |

Numbers are n(%)

* Coagulase-negative staphylococci were considered contaminants if reported as such after clinical consultation of the microbiology staff.
