# Supplementary material for: Low frequency of community-acquired bacterial co-infection in patients hospitalized for COVID-19 based on clinical, radiological and microbiological criteria: a retrospective cohort study
Source: Antimicrob Resist Infect Control. 2021 Oct 30;10:155. doi: 10.1186/s13756-021-01024-4 (PMC8556861; doi:10.1186/s13756-021-01024-4)
Supplement: Supplementary file 3 — Additional file 3. Description of criteria present in patients with a possible or probable co-infection. [file 13756_2021_1024_MOESM3_ESM.docx]

**Additional file 3. Description of criteria present in patients with a possible or probable co-infection.**

|  | **Clinical parameters present** | **CT findings present** | **Microbiologal results positive** | **1 criterion** | **≥ 1 criterion** |
| --- | --- | --- | --- | --- | --- |
| Probable (n = 3) | 3/3 | 2/3 | 2/3 | - | 3/3 |
| Possible (n = 35) | 19/35 | 7/35 | 9/35 | 35/35 | - |

CT = computed tomography.
